# Crystal structure, Hirshfeld surface analysis and inter­action energy and DFT studies of methyl 4-[3,6-bis­(pyridin-2-yl)pyridazin-4-yl]benzoate

**DOI:** 10.1107/S2056989019013732

**Published:** 2019-10-22

**Authors:** Mouad Filali, Lhoussaine El Ghayati, Tuncer Hökelek, Joel T. Mague, Abdessalam Ben-Tama, El Mestafa El Hadrami, Nada Kheira Sebbar

**Affiliations:** a Laboratoire de Chimie Organique Appliquée, Université Sidi Mohamed Ben Abdallah, Faculté des Sciences et Techniques, Route d’immouzzer, BP 2202, Fez, Morocco; b Laboratoire de Chimie Organique Hétérocyclique URAC 21, Pôle de Compétence Pharmacochimie, Av. Ibn Battouta, BP 1014, Faculté des Sciences, Université Mohammed V, Rabat, Morocco; c Department of Physics, Hacettepe University, 06800 Beytepe, Ankara, Turkey; d Department of Chemistry, Tulane University, New Orleans, LA 70118, USA; e Laboratoire de Chimie Appliquée et Environnement, Equipe de Chimie Bioorganique Appliquée, Faculté des sciences, Université Ibn Zohr, Agadir, Morocco

**Keywords:** crystal structure, pyridazine, pyridine, hydrogen bond, C—H⋯π(ring) inter­action, Hirshfeld surface

## Abstract

The title com­pound, C_22_H_16_N_4_O_2_, contains two pyridine rings and one meth­oxy­carbonyl­phenyl group attached to a pyridazine ring which deviates very slightly from planarity. In the crystal, ribbons consisting of inversion-related chains of mol­ecules extending along the *a*-axis direction are formed by C—H_Mthy_⋯O_Carbx_ (Mthy = methyl and Carbx = carboxyl­ate) hydrogen bonds. The ribbons are connected into layers parallel to the *bc* plane by C—H_Bnz_⋯π(ring) (Bnz = benzene) inter­actions. The Hirshfeld surface analysis of the crystal structure indicates that the most important contributions for the crystal packing are from H⋯H (39.7%), H⋯C/C⋯H (27.5%), H⋯N/N⋯H (15.5%) and O⋯H/H⋯O (11.1%) inter­actions. Hydrogen-bonding and van der Waals inter­actions are the dominant inter­actions in the crystal packing. Computational chemistry indicates that in the crystal, C—H_Mthy_⋯O_Carbx_ hydrogen-bond energies are 62.0 and 34.3 kJ mol^−1^, respectively. Density functional theory (DFT) optimized structures at the B3LYP/6-311G(d,p) level are com­pared with the experimentally determined mol­ecular structure in the solid state. The HOMO–LUMO behaviour was elucidated to determine the energy gap.

## Abstract 2

The pyridazine ring deviates slightly from planarity. In the crystal, ribbons consisting of inversion-related chains of mol­ecules extending along the *a*-axis direction are formed by C—H_Mthy_⋯O_Carbx_ (Mthy = methyl and Carbx = carboxyl­ate) hydrogen bonds. The ribbons are connected into layers parallel to the *bc* plane by inversion-related C—H_Bnz_⋯π(ring) inter­actions.

## Chemical context   

3,6-Bis(pyridin-2-yl)pyridazine derivatives are a versatile class of nitro­gen-containing heterocyclic com­pounds and they constitute useful inter­mediates in organic syntheses. Also, this nucleus is one of the important ligands in the field of coordination chemistry research. 5-[3,6-Bis(pyridin-2-yl)pyri­da­zine-4-yl]-2′-de­oxy­uridine-5′-*O*-triphosphate can be used as a potential substrate for fluorescence detection and imaging of DNA (Kore *et al.*, 2015 ▸). Systems containing this moiety also showed remarkable corrosion inhibition (Khadiri *et al.*, 2016 ▸). Heterocyclic mol­ecules such as 3,6-bis­(pyridin-2-yl)-1,2,4,5-tetra­zine have been used in transition-metal chemistry (Kaim & Kohlmann, 1987 ▸); this tetrazine is a bidentate chelating ligand popular in coordination chemistry and com­plexes of a wide range of metals, including iridium and palladium (Tsukada *et al.*, 2001 ▸). As a continuation of our research in the field of substituted 3,6-bis­(pyridin-2-yl)pyridazine (Filali *et al.*, 2019*a* ▸,*b* ▸), we report herein the synthesis, the mol­ecular and crystal structures, along with the Hirshfeld surface analysis, the inter­molecular inter­action energies and the density functional theory (DFT) com­putational calculations carried out at the B3LYP/6-311G(d,p) level for a new 3,6-bis­(pyridin-2-yl)pyridazine, namely, methyl 4-[3,6-bis­(pyridin-2-yl)pyridazin-4-yl]benzoate, (I).
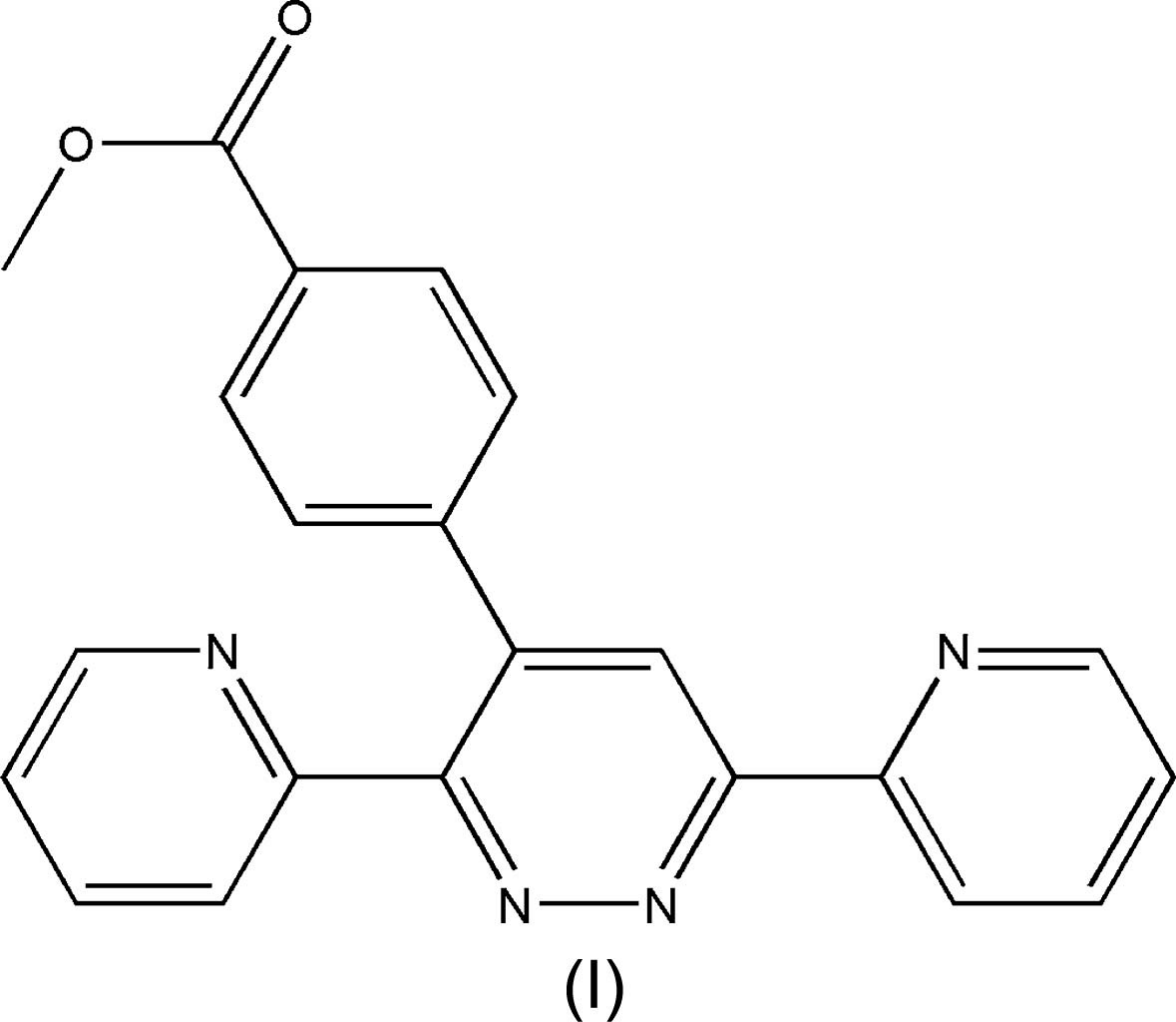


## Structural commentary   

**Figure 1:**
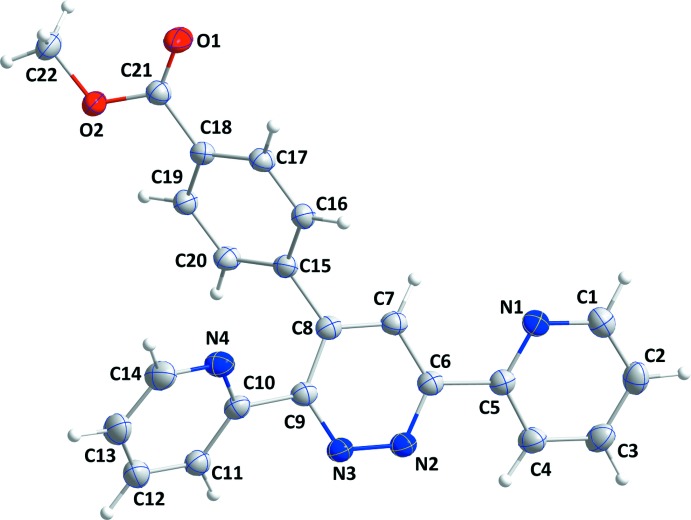
The mol­ecular structure of the title com­pound with the atom-numbering scheme. Displacement ellipsoids are drawn at the 50% probability level.

The title com­pund contains two pyridine rings and one meth­oxy­carbonyl­phenyl group attached to a pyridazine ring, where the central pyridazine ring, *B* (atoms N2/N3/C6–C9), deviates slightly from planarity by ±0.021 (1) Å (r.m.s. deviation = 0.0134 Å) (Fig. 1 ▸). The planes of the pyridine rings, *A* (N1/C1–C5) and *C* (N4/C10–C14), are inclined to the mean plane of the pyridazine ring, *B*, by 18.68 (6) and 38.40 (6)°, respectively, while the benzene ring, *D* (C15–C20), is inclined to it by 62.59 (5)°. The pyridine and benzene rings are oriented at dihedral angles of *A*/*C* = 25.16 (4)°, *A*/*D* = 48.94 (4)° and *C*/*D* = 59.13 (4)°. The plane of the carboxyl group (defined by atoms C18/C21/O1/O2) is twisted out of the plane of the benzene ring, *D*, by 22.88 (5)°.

## Supra­molecular features   

**Figure 2:**
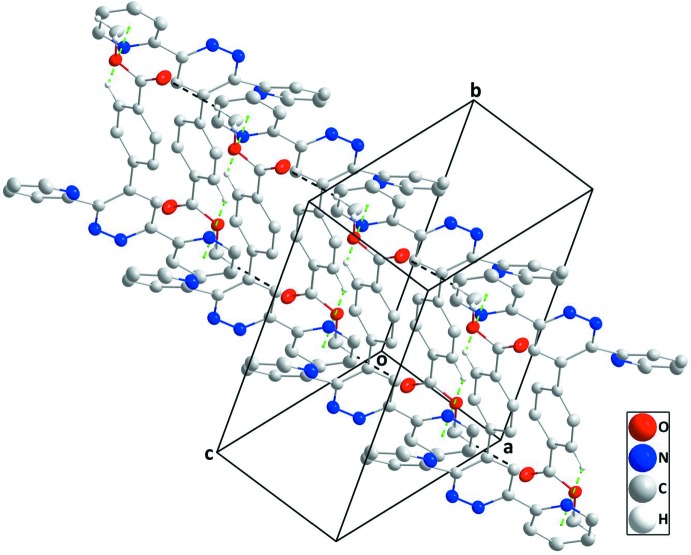
A partial packing diagram showing two chains connnected by C—H⋯π(ring) inter­actions (green dashed lines). The C—H_Mthy_⋯O_Carbx_ (Mthy = methyl and Carbx = carboxyl­ate) hydrogen bonds are shown as black dashed lines.

In the crystal, chains of mol­ecules extending along the *a*-axis direction are formed by C22—H22*C*⋯O1^v^ hydrogen bonds (Table 1 ▸). Inversion-related chains are connected into ribbons by C22—H22*B*⋯O1^iv^ hydrogen bonds (Table 1 ▸) and the ribbons are joined into stepped layers approximately parallel to (01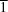) by inversion-related pairs of C19—H19⋯*Cg*1^i^ inter­actions, where *Cg*1 is the centroid of pyridine ring *A* (Table 1 ▸ and Fig. 2 ▸). The Hirshfeld surface analysis of the crystal structure indicates that the most important contributions for the crystal packing are from H⋯H (39.7%), H⋯C/C⋯H (27.5%), H⋯N/N⋯H (15.5%) and O⋯H/H⋯O (11.1%) inter­actions. Hydrogen-bonding and van der Waals inter­actions are the dominant inter­actions in the crystal packing.

## Hirshfeld surface analysis   

**Figure 3:**
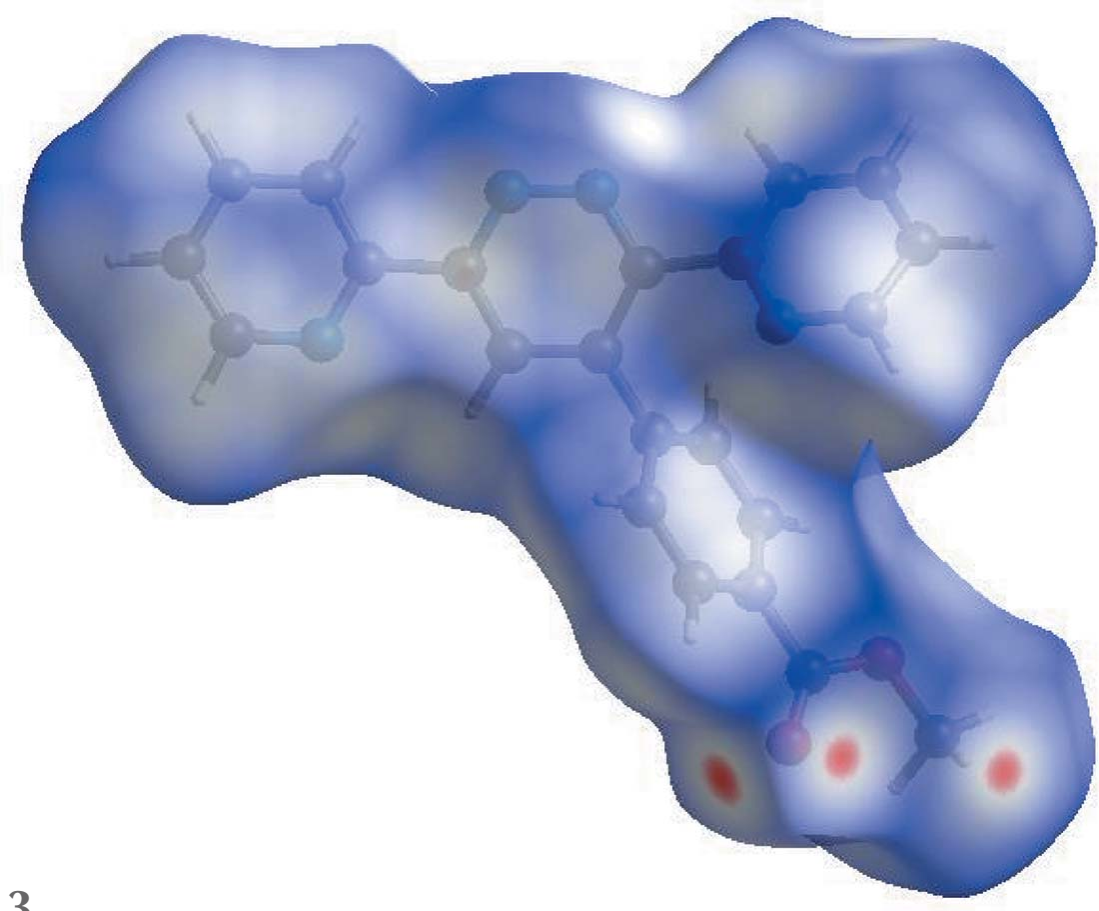
View of the three-dimensional Hirshfeld surface of the title com­pound plotted over *d*_norm_ in the range −0.1417 to 1.3796 a.u.

**Figure 4:**
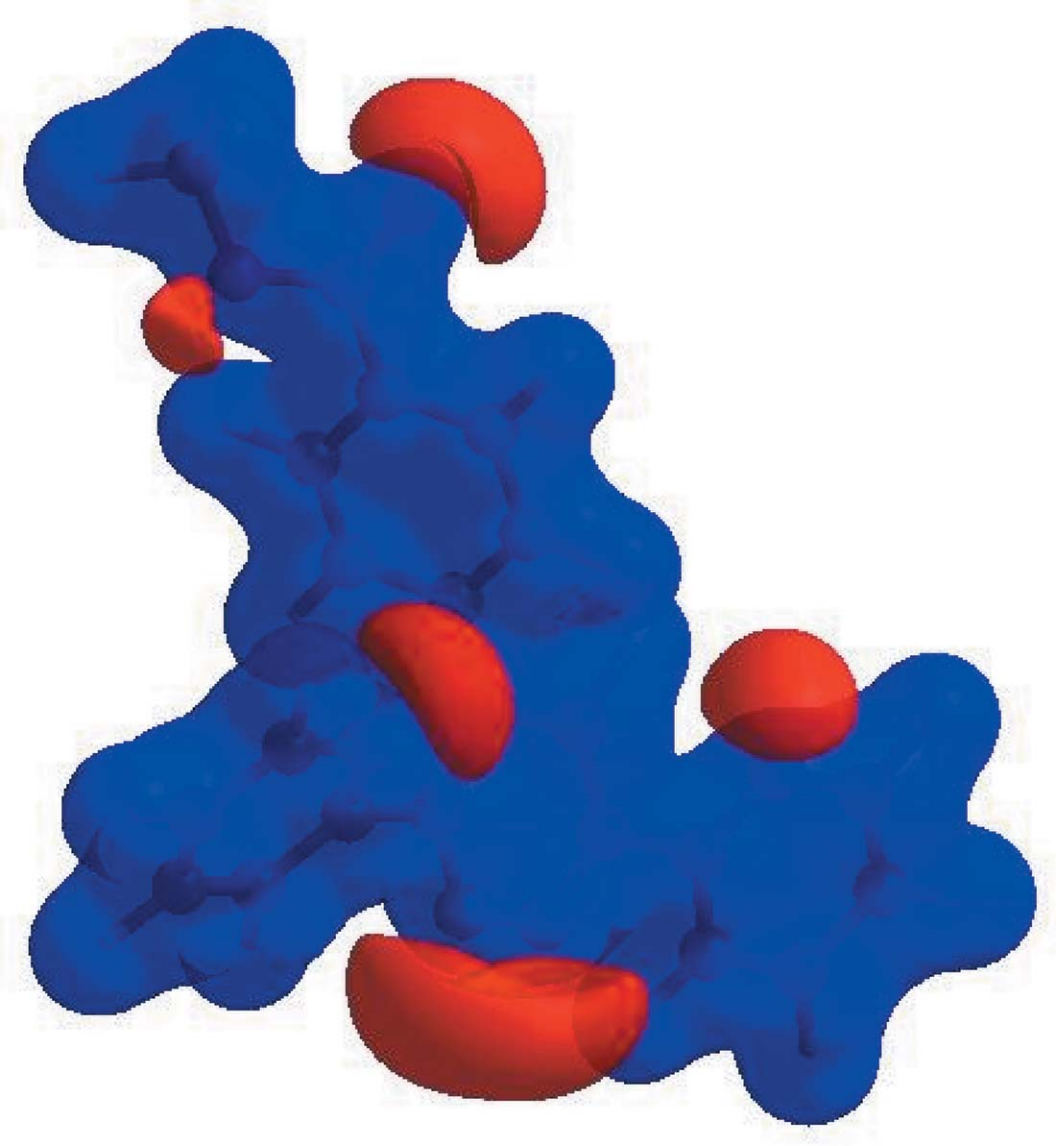
View of the three-dimensional Hirshfeld surface of the title com­pound plotted over the electrostatic potential energy in the range −0.0500 to 0.0500 a.u. using the STO-3G basis set at the Hartree–Fock level of theory. Hydrogen-bond donors and acceptors are shown as blue and red regions around the atoms corresponding to positive and negative potentials, respectively.

**Figure 5:**
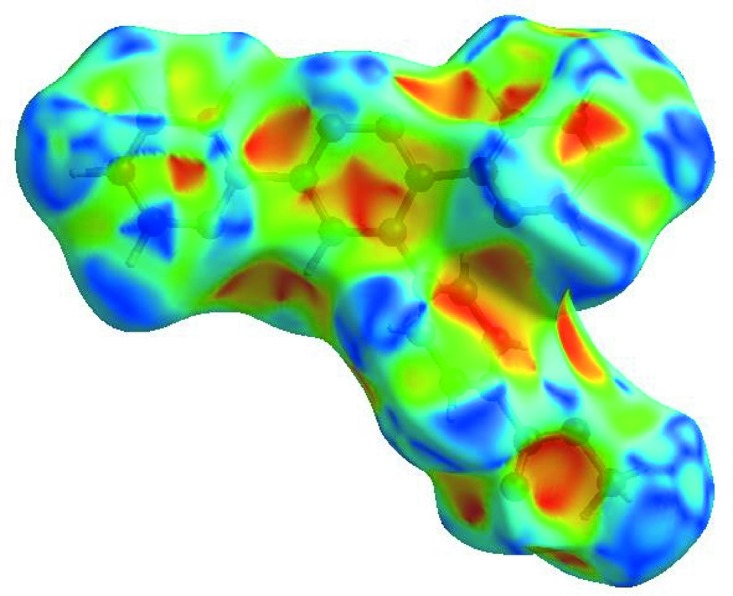
Hirshfeld surface of the title com­pound plotted over shape-index.

**Figure 6:**
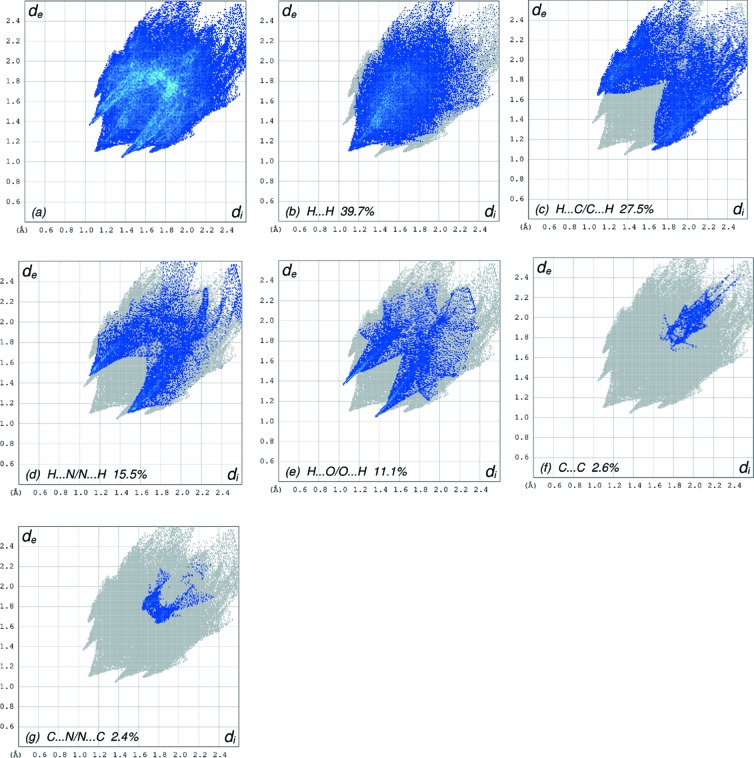
The full two-dimensional fingerprint plots for the title com­pound, showing (*a*) all inter­actions, and delineated into (*b*) H⋯H, (*c*) H⋯C/C⋯H, (*d*) H⋯N/N⋯H, (*e*) H⋯O/O⋯H, (*f*) C⋯C and (*g*) C⋯N/N⋯C inter­actions. The *d*_i_ and *d*_e_ values are the closest inter­nal and external distances (in Å) from given points on the Hirshfeld surface contacts.

In order to visualize the inter­molecular inter­actions in the crystal of the title com­pound, a Hirshfeld surface (HS) analysis (Hirshfeld, 1977 ▸; Spackman & Jayatilaka, 2009 ▸) was carried out by using *CrystalExplorer* (Version 17.5; Turner *et al.*, 2017 ▸). In the HS plotted over *d*_norm_ (Fig. 3 ▸), the white surface indicates contacts with distances equal to the sum of the van der Waals radii, and the red and blue colours indicate distances shorter (in close contact) or longer (distinct contact) than the van der Waals radii, respectively (Venkatesan *et al.*, 2016 ▸). The bright-red spots appearing near atoms O1 and H22*B* and H22*C* indicate their roles as the respective donors and/or acceptors; they also appear as blue and red regions corresponding to positive and negative potentials on the HS mapped over electrostatic potential (Spackman *et al.*, 2008 ▸; Jayatilaka *et al.*, 2005 ▸), as shown in Fig. 4 ▸. The blue regions indicate the positive electrostatic potential (hydrogen-bond donors), while the red regions indicate the negative electrostatic potential (hydrogen-bond acceptors). The shape-index of the HS is a tool to visualize the π–π stacking by the presence of adjacent red and blue triangles; if there are no adjacent red and/or blue triangles, then there are no π–π inter­actions. Fig. 5 ▸ clearly suggests that there are no π—π inter­actions in (I). The overall two-dimensional fingerprint plot (Fig. 6 ▸ *a*) and those delineated into H⋯H, H⋯C/C⋯H, H⋯N/N⋯H, H⋯O/O⋯H, C⋯C and C⋯N/N⋯C contacts (McKinnon *et al.*, 2007 ▸) are illustrated in Figs. 6 ▸ (*b*)–(*g*), respectively, together with their relative contributions to the Hirshfeld surface. The most important inter­action is H⋯H contributing 39.7% to the overall crystal packing, which is reflected in Fig. 6 ▸(*b*) as widely scattered points of high density due to the large hydrogen content of the mol­ecule with the tip at *d*_e_ = *d*_i_ = 1.10 Å, due to the short inter­atomic H⋯H contacts (Table 2 ▸). Due to the presence of C—H⋯π inter­actions, a 27.5% contribution to the HS arises from the H⋯C/C⋯H contacts (Table 2 ▸) which are viewed as pairs of spikes in the fingerprint plot shown in Fig. 6 ▸(*c*) with the tips at *d*_e_ + *d*_i_ = 2.75 Å. The pair of scattered points of wings resulting in the fingerprint plots delineated into H⋯N/N⋯H (Fig. 6 ▸ *d*) contacts, with a 15.5% contribution to the HS, has a symmetrical distribution of points with the edges at *d*_e_ + *d*_i_ = 2.58 Å (Table 2 ▸). The pair of characteristic wings resulting in the fingerprint plot shown in Fig. 6 ▸(*e*), with an 11.1% contribution to the HS, arises from the O⋯H/H⋯O contacts (Table 2 ▸) and is viewed as pair of spikes with the tips at *d*_e_ + *d*_i_ = 2.42 Å. The C⋯C contacts (Fig. 6 ▸ *f*) have an arrow-shaped distribution of points with the tip at *d*_e_ = *d*_i_ = 13.50 Å. Finally, the tiny characteristic wings resulting in the fingerprint plots shown in Fig. 6 ▸ *g*, a 2.4% contribution to the HS, arises from the C⋯N/N⋯C contacts (Table 2 ▸) and is viewed with the tip at *d*_e_ = *d*_i_ = 3.40 Å.

**Figure 7:**
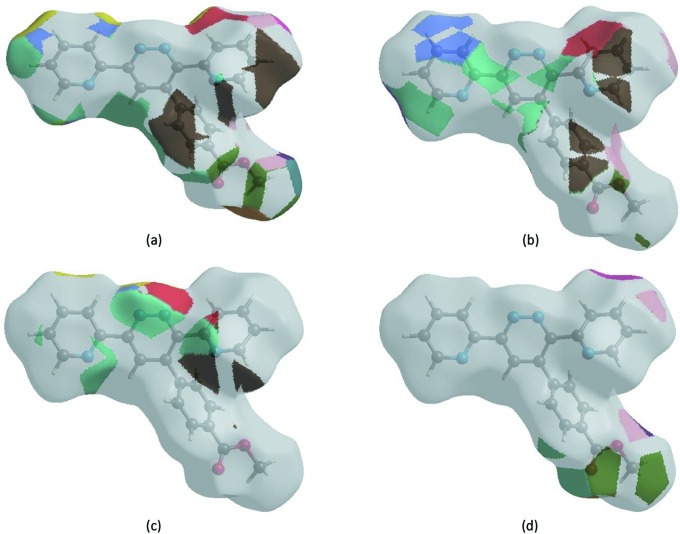
The Hirshfeld surface representations with the function *d*_norm_ plotted onto the surface for (*a*) H⋯H, (*b*) H⋯C/C⋯H, (*c*) H⋯N/N⋯H and (*d*) H⋯O/O⋯H inter­actions.

The Hirshfeld surface representations with the function *d*_norm_ plotted onto the surface are shown for the H⋯H, H⋯C/C⋯H, H⋯N/N⋯H and H⋯O/O⋯H inter­actions in Figs. 7 ▸(*a*)–(*d*), respectively.

The Hirshfeld surface analysis confirms the importance of H-atom contacts in establishing the packing. The large number of H⋯H, H⋯C/C⋯H, H⋯N/N⋯H and H⋯O/O⋯H inter­actions suggest that van der Waals inter­actions and hydrogen bonding play the major roles in the crystal packing (Hathwar *et al.*, 2015 ▸).

## Inter­action energy calculations   

The inter­molecular inter­action energies were calculated using the CE–B3LYP/6-31G(d,p) energy model available in *CrystalExplorer* (CE) (Version 17.5; Turner *et al.*, 2017 ▸), where a cluster of mol­ecules would need to be generated by applying crystallographic symmetry operations with respect to a selected central mol­ecule within a default radius of 3.8 Å (Turner *et al.*, 2014 ▸). The total inter­molecular energy (*E*_tot_) is the sum of the electrostatic (*E*_ele_), polarization (*E*_pol_), dispersion (*E*_dis_) and exchange–repulsion (*E*_rep_) energies (Turner *et al.*, 2015 ▸), with scale factors of 1.057, 0.740, 0.871 and 0.618, respectively (Mackenzie *et al.*, 2017 ▸). Hydrogen-bonding inter­action energies (in kJ mol^−1^) were calculated as −23.9 (*E*_ele_), −4.3 (*E*_pol_), −76.2 (*E*_dis_), 53.0 (*E*_rep_) and −62.0 (*E*_tot_) for the C22—H22*C*⋯O1 hydrogen-bonding inter­action, and −22.0 (*E*_ele_), −8.5 (*E*_pol_), −28.5 (*E*_dis_), 32.3 (*E*_rep_) and −34.3 (*E*_tot_) for the C22—H22*B*⋯O1 hydrogen-bonding inter­action.

## DFT calculations   

**Figure 8:**
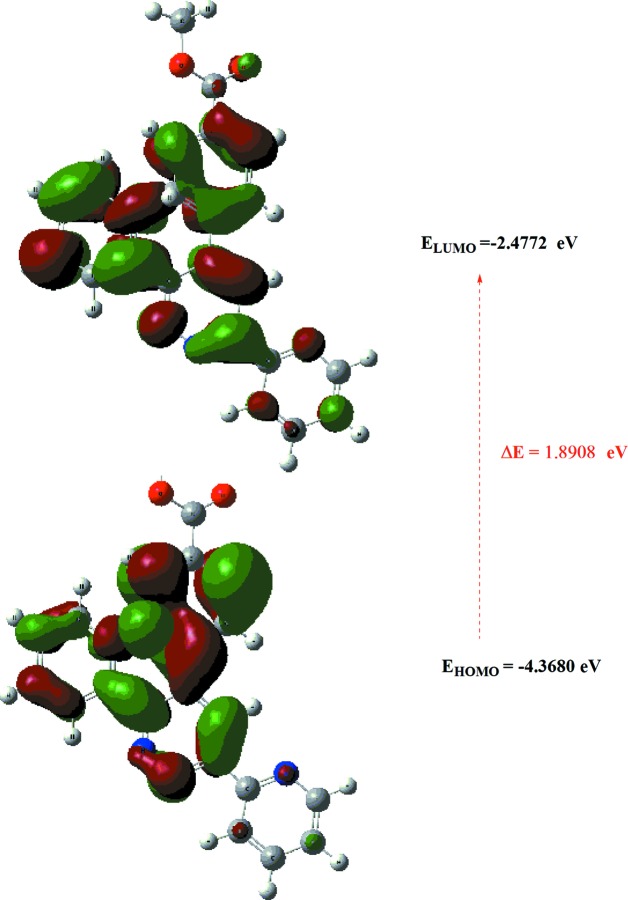
The energy band gap of the title com­pound.

The optimized structure of the title com­pound, (I), in the gas phase was generated theoretically *via* density functional theory (DFT) using the standard B3LYP functional and 6-311G(d,p) basis-set calculations (Becke, 1993 ▸) as implemented in *GAUSSIAN09* (Frisch *et al.*, 2009 ▸). The theoretical and experimental results are in good agreement (Table 3 ▸). The highest-occupied mol­ecular orbital (HOMO), acting as an electron donor, and the lowest-unoccupied mol­ecular orbital (LUMO), acting as an electron acceptor, are very important parameters for quantum chemistry. When the energy gap is small, the mol­ecule is highly polarizable and has high chemical reactivity. The DFT calculations provide some important information on the reactivity and site selectivity of the mol­ecular framework. *E*_HOMO_ and *E*_LUMO_ clarify the inevitable charge exchange collaboration inside the studied material, and electronegativity (χ), hardness (η), potential (μ), electrophilicity (ω) and softness (σ) are all recorded in Table 4 ▸. The significance of η and σ is to evaluate both the reactivity and stability. The electron transition from the HOMO to the LUMO energy level is shown in Fig. 8 ▸. The HOMO and LUMO are localized in the plane extending from the whole methyl 4-[3,6-bis­(pyridin-2-yl)pyridazin-4-yl]benzoate ring. The energy band gap [Δ*E* = *E*_LUMO_ − *E*_HOMO_] of the mol­ecule is about 1.8908 eV, and the frontier mol­ecular orbital (FMO) energies, *i.e. E*_HOMO_ and *E*_LUMO_, are −4.3680 and −2.4772 eV, respectively.

## Database survey   

A 4-[(prop-2-en-1-yl­oxy)meth­yl]phenyl analogue has been reported (Filali *et al.*, 2019*a* ▸). Three other metal com­plexes coordinated by 3,6-bis­(pyridin-2-yl)pyridazine have also been reported, namely aqua­bis­[3,6-bis­(pyridin-2-yl)pyridazine-κ^2^ *N* ^1^,*N* ^6^]copper(II) bis­(tri­fluoro­methane­sulfonate) (Showrilu *et al.*, 2017 ▸), tetra­kis­[μ-3,6-di(pyridin-2-yl)pyridazine]bis­(μ-hydroxo)bis­(μ-aqua)­tetra­nickel(II) hexa­nitrate tetra­deca­hydrate (Marino *et al.*, 2019 ▸) and *catena*-[[μ_2_-3,6-bis(pyridin-2-yl)pyridazine]bis­(μ-2-azido)­dizaidodicopper monohydrate] (Mastropietro *et al.*, 2013 ▸).

## Synthesis and crystallization   

3,6-Bis(pyridin-2-yl)-1,2,4,5-tetra­zine (4 mmol) was dissolved in toluene (20 ml), and then 1 equiv. of methyl 4-ethynylbenzoate was added and the reaction mixture was stirred and refluxed at temperatures between 413 and 453 K. The solvent was then evaporated. The product obtained was separated by chromatography on a column of silica gel. The isolated solid was recrystallized from hexa­ne–di­chloro­methane (1:1 *v*/*v*) to afford colourless crystals (yield 92%; m.p. 449 K).

## Refinement   

The experimental details including the crystal data, data collection and refinement are summarized in Table 5 ▸. H atoms were located in a difference Fourier map and refined freely.

## Supplementary Material

Crystal structure: contains datablock(s) I, global. DOI: 10.1107/S2056989019013732/lh5927sup1.cif

Structure factors: contains datablock(s) I. DOI: 10.1107/S2056989019013732/lh5927Isup2.hkl

Supporting information file. DOI: 10.1107/S2056989019013732/lh5927Isup3.cdx

Supporting information file. DOI: 10.1107/S2056989019013732/lh5927Isup4.cml

CCDC references: 1958277, 1958277

Additional supporting information:  crystallographic information; 3D view; checkCIF report

## Figures and Tables

**Table 1:** Hydrogen-bond geometry (Å, °) *Cg*1 is the centroid of pyridyl ring *A* (atoms N1/C1–C5).

*D*—H⋯*A*	*D*—H	H⋯*A*	*D*⋯*A*	*D*—H⋯*A*
C19—H19⋯*Cg*1^i^	0.967 (15)	2.876 (15)	3.5715 (13)	129.8 (12)
C22—H22*B*⋯O1^iv^	0.964 (19)	2.598 (19)	3.5407 (19)	165.8 (14)
C22—H22*C*⋯O1^v^	0.959 (19)	2.536 (19)	3.4924 (16)	174.8 (15)

Symmetry codes: (i) 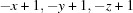; (iv) 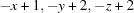; (v) 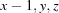.

**Table 2:** Selected interatomic distances (Å)

O2⋯C3^i^	3.4089 (16)	N4⋯H14^viii^	2.931 (18)
O1⋯H12^ii^	2.765 (16)	C1⋯C6^iii^	3.4447 (17)
O1⋯H22*A*	2.573 (18)	C1⋯C7^iii^	3.5272 (17)
O1⋯H22*B*	2.660 (18)	C2⋯C17^ix^	3.4292 (18)
O1⋯H22*C* ^iii^	2.537 (19)	C3⋯C3^x^	3.5516 (18)
O1⋯H22*B* ^iv^	2.598 (18)	C6⋯C11^iii^	3.3751 (16)
O1⋯H17	2.611 (14)	C10⋯C20	3.3022 (16)
O2⋯H17^v^	2.773 (13)	C11⋯C22^vii^	3.4630 (18)
O2⋯H19	2.462 (14)	C14⋯C17^viii^	3.4960 (18)
N1⋯C9^iii^	3.4168 (15)	C14⋯C16^viii^	3.4987 (18)
N2⋯C1^v^	3.4404 (16)	C1⋯H19^i^	2.926 (16)
N4⋯C20	3.2459 (16)	C6⋯H11^iii^	2.924 (15)
N4⋯C15	2.8825 (15)	C7⋯H1^v^	2.988 (17)
N4⋯C16	3.4362 (16)	C11⋯H22*A* ^vii^	2.964 (18)
N1⋯H20^iii^	2.750 (15)	C16⋯H14^viii^	2.891 (17)
N1⋯H7	2.553 (14)	C17⋯H2^ix^	2.898 (18)
N2⋯H4^vi^	2.710 (17)	C18⋯H2^ix^	2.822 (18)
N2⋯H4	2.511 (17)	H3⋯H11^vi^	2.53 (2)
N3⋯H11	2.644 (13)	H4⋯H4^vi^	2.43 (3)
N3⋯H22*A* ^vii^	2.713 (17)	H7⋯H20^iii^	2.43 (2)
N3⋯H3^vi^	2.644 (18)	H16⋯H19^iii^	2.57 (2)

Symmetry codes: (i) 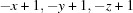; (ii) 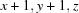; (iii) 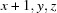; (iv) 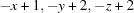; (v) 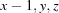; (vi) 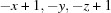; (vii) 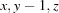; (viii) 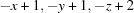; (ix) 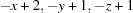; (x) 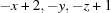.

**Table 3:** Comparison of selected (X-ray and DFT) geometric data (Å, °)

Bonds/angles	X-ray	B3LYP/6-311G(d,p)
O1—C21	1.2049 (14)	1.23831
O2—C21	1.3342 (15)	1.38677
O2—C22	1.4501 (14)	1.45892
N1—C1	1.3411 (16)	1.38974
N1—C5	1.3461 (15)	1.40690
N2—C6	1.3366 (15)	1.36917
N2—N3	1.3407 (14)	1.31753
N3—C9	1.3375 (14)	1.38785
N4—C14	1.3388 (17)	1.34410
N4—C10	1.3429 (15)	1.35601
C21—O2—C22	115.72 (9)	116.46416
C1—N1—C5	116.73 (10)	117.59335
C6—N2—N3	119.41 (9)	118.73596
C9—N3—N2	120.51 (9)	121.63356
C14—N4—C10	117.22 (11)	118.30113
N1—C5—C4	123.17 (11)	123.94848
N1—C5—C6	115.74 (10)	116.62957
N2—C6—C7	122.43 (10)	122.86465
N2—C6—C5	115.74 (10)	115.11012

**Table 4:** Calculated energies

Mol­ecular Energy (a.u.) (eV)	Compound (I)
Total Energy *TE* (eV)	−33114.5851
*E*_HOMO_ (eV)	−4.3680
*E*_LUMO_ (eV)	−2.4772
Gap *ΔE* (eV)	1.8908
Dipole moment *μ* (Debye)	5.0683
Ionization potential *I* (eV)	4.3680
Electron affinity *A*	2.4772
Electronegativity *χ*	3.4226
Hardness *η*	0.9454
Electrophilicity index *ω*	6.1953
Softness *σ*	1.0577
Fraction of electron transferred *ΔN*	1.8920

**Table 5:** Experimental details

Crystal data	
Chemical formula	C_22_H_16_N_4_O_2_
*M*_r_	368.39
Crystal system, space group	Triclinic, *P* 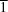
Temperature (K)	150
*a*, *b*, *c* (Å)	6.0464 (1), 11.7175 (3), 13.2931 (3)
α, β, γ (°)	95.735 (1), 95.813 (1), 101.780 (1)
*V* (Å^3^)	910.16 (3)
*Z*	2
Radiation type	Cu *K*α
μ (mm^−1^)	0.72
Crystal size (mm)	0.26 × 0.12 × 0.07

Data collection	
Diffractometer	Bruker D8 VENTURE PHOTON 100 CMOS
Absorption correction	Multi-scan (*SADABS*; Krause *et al.*, 2015 ▸)
*T*_min_, *T*_max_	0.85, 0.95
No. of measured, independent and observed [*I* > 2σ(*I*)] reflections	7056, 3426, 3139
*R*_int_	0.022
(sin θ/λ)_max_ (Å^−1^)	0.625

Refinement	
*R*[*F* ^2^ > 2σ(*F* ^2^)], *wR*(*F* ^2^), *S*	0.036, 0.098, 1.02
No. of reflections	3426
No. of parameters	318
H-atom treatment	All H-atom parameters refined
Δρ_max_, Δρ_min_ (e Å^−3^)	0.23, −0.17

Computer programs: *APEX3* (Bruker, 2016 ▸), *SAINT* (Bruker, 2016 ▸), *SAINT* (Bruker, 2016 ▸), *SHELXT* (Sheldrick, 2015*a* ▸), *SHELXL2018* (Sheldrick, 2015*b* ▸), *DIAMOND* (Brandenburg & Putz, 2012 ▸) and *SHELXTL* (Bruker, 2016 ▸).

## supplementary crystallographic information

### Crystal data

**Table:**

C_22_H_16_N_4_O_2_	*Z* = 2
*M**_r_* = 368.39	*F*(000) = 384
Triclinic, *P*1	*D*_x_ = 1.344 Mg m^−^^3^
*a* = 6.0464 (1) Å	Cu *K*α radiation, λ = 1.54178 Å
*b* = 11.7175 (3) Å	Cell parameters from 6104 reflections
*c* = 13.2931 (3) Å	θ = 3.4–74.7°
α = 95.735 (1)°	µ = 0.72 mm^−^^1^
β = 95.813 (1)°	*T* = 150 K
γ = 101.780 (1)°	Column, colourless
*V* = 910.16 (3) Å^3^	0.26 × 0.12 × 0.07 mm

### Data collection

**Table:**

Bruker D8 VENTURE PHOTON 100 CMOS diffractometer	3426 independent reflections
Radiation source: INCOATEC IµS micro-focus source	3139 reflections with *I* > 2σ(*I*)
Mirror monochromator	*R*_int_ = 0.022
Detector resolution: 10.4167 pixels mm^-1^	θ_max_ = 74.7°, θ_min_ = 3.4°
ω scans	*h* = −6→7
Absorption correction: multi-scan (*SADABS*; Krause *et al.*, 2015)	*k* = −14→13
*T*_min_ = 0.85, *T*_max_ = 0.95	*l* = −14→16
7056 measured reflections	

### Refinement

**Table:**

Refinement on *F*^2^	Secondary atom site location: difference Fourier map
Least-squares matrix: full	Hydrogen site location: difference Fourier map
*R*[*F*^2^ > 2σ(*F*^2^)] = 0.036	All H-atom parameters refined
*wR*(*F*^2^) = 0.098	*w* = 1/[σ^2^(*F*_o_^2^) + (0.0539*P*)^2^ + 0.2048*P*] where *P* = (*F*_o_^2^ + 2*F*_c_^2^)/3
*S* = 1.02	(Δ/σ)_max_ < 0.001
3426 reflections	Δρ_max_ = 0.23 e Å^−^^3^
318 parameters	Δρ_min_ = −0.16 e Å^−^^3^
0 restraints	Extinction correction: *SHELXL2018* (Sheldrick, 2015*b*), Fc^*^=kFc[1+0.001xFc^2^λ^3^/sin(2θ)]^-1/4^
Primary atom site location: dual space	Extinction coefficient: 0.0087 (8)

### Special details

**Table:**

Geometry. All esds (except the esd in the dihedral angle between two l.s. planes) are estimated using the full covariance matrix. The cell esds are taken into account individually in the estimation of esds in distances, angles and torsion angles; correlations between esds in cell parameters are only used when they are defined by crystal symmetry. An approximate (isotropic) treatment of cell esds is used for estimating esds involving l.s. planes.
Refinement. Refinement of F^2^ against ALL reflections. The weighted R-factor wR and goodness of fit S are based on F^2^, conventional R-factors R are based on F, with F set to zero for negative F^2^. The threshold expression of F^2^ > 2sigma(F^2^) is used only for calculating R-factors(gt) etc. and is not relevant to the choice of reflections for refinement. R-factors based on F^2^ are statistically about twice as large as those based on F, and R- factors based on ALL data will be even larger.

### Fractional atomic coordinates and isotropic or equivalent isotropic displacement parameters (Å^2^)

**Table:**

	*x*	*y*	*z*	*U*_iso_*/*U*_eq_	
O1	0.62576 (15)	0.94764 (7)	0.85874 (8)	0.0425 (3)	
O2	0.24890 (14)	0.87662 (7)	0.83965 (7)	0.0313 (2)	
N1	1.02078 (16)	0.30995 (9)	0.51060 (7)	0.0290 (2)	
N2	0.52919 (17)	0.16735 (8)	0.61309 (8)	0.0294 (2)	
N3	0.38764 (17)	0.18823 (8)	0.68071 (8)	0.0292 (2)	
N4	0.32230 (18)	0.39009 (9)	0.87963 (8)	0.0328 (2)	
C1	1.1730 (2)	0.28112 (11)	0.45151 (9)	0.0319 (3)	
H1	1.308 (3)	0.3455 (14)	0.4491 (12)	0.041 (4)*	
C2	1.1492 (2)	0.17134 (12)	0.39688 (10)	0.0352 (3)	
H2	1.270 (3)	0.1577 (15)	0.3566 (14)	0.053 (5)*	
C3	0.9553 (2)	0.08688 (11)	0.40089 (10)	0.0361 (3)	
H3	0.929 (3)	0.0079 (15)	0.3616 (13)	0.047 (4)*	
C4	0.7948 (2)	0.11428 (11)	0.46092 (9)	0.0325 (3)	
H4	0.656 (3)	0.0587 (15)	0.4647 (12)	0.047 (4)*	
C5	0.83537 (19)	0.22568 (10)	0.51552 (8)	0.0263 (2)	
C6	0.67584 (19)	0.25744 (10)	0.58616 (8)	0.0258 (2)	
C7	0.67982 (19)	0.37445 (10)	0.62177 (9)	0.0264 (2)	
H7	0.783 (2)	0.4381 (13)	0.5971 (11)	0.031 (3)*	
C8	0.53152 (19)	0.39724 (9)	0.68926 (8)	0.0253 (2)	
C9	0.38988 (19)	0.29823 (10)	0.71976 (8)	0.0257 (2)	
C10	0.2376 (2)	0.30746 (9)	0.79999 (9)	0.0267 (2)	
C11	0.0258 (2)	0.23148 (10)	0.79314 (9)	0.0297 (3)	
H11	−0.027 (3)	0.1701 (13)	0.7335 (12)	0.037 (4)*	
C12	−0.1065 (2)	0.24211 (11)	0.87157 (10)	0.0356 (3)	
H12	−0.260 (3)	0.1895 (14)	0.8668 (12)	0.042 (4)*	
C13	−0.0224 (3)	0.32801 (12)	0.95334 (11)	0.0398 (3)	
H13	−0.111 (3)	0.3385 (15)	1.0109 (14)	0.050 (4)*	
C14	0.1922 (2)	0.39898 (11)	0.95456 (10)	0.0386 (3)	
H14	0.258 (3)	0.4584 (14)	1.0115 (13)	0.042 (4)*	
C15	0.51538 (18)	0.51941 (9)	0.72438 (8)	0.0245 (2)	
C16	0.70032 (19)	0.59753 (10)	0.78141 (9)	0.0264 (2)	
H16	0.839 (2)	0.5708 (12)	0.7993 (11)	0.032 (3)*	
C17	0.68255 (19)	0.71046 (10)	0.81609 (9)	0.0273 (3)	
H17	0.807 (2)	0.7636 (13)	0.8570 (11)	0.033 (4)*	
C18	0.47906 (18)	0.74593 (9)	0.79418 (8)	0.0247 (2)	
C19	0.29575 (19)	0.66908 (10)	0.73420 (9)	0.0277 (3)	
H19	0.157 (3)	0.6959 (12)	0.7180 (11)	0.032 (4)*	
C20	0.3147 (2)	0.55664 (10)	0.69887 (9)	0.0290 (3)	
H20	0.188 (3)	0.5009 (13)	0.6556 (12)	0.039 (4)*	
C21	0.46364 (19)	0.86730 (10)	0.83384 (9)	0.0271 (3)	
C22	0.2179 (2)	0.99301 (11)	0.87423 (12)	0.0371 (3)	
H22A	0.289 (3)	1.0486 (15)	0.8283 (13)	0.046 (4)*	
H22B	0.287 (3)	1.0160 (15)	0.9439 (14)	0.050 (5)*	
H22C	0.056 (3)	0.9847 (15)	0.8673 (13)	0.051 (5)*	

### Atomic displacement parameters (Å^2^)

**Table:**

	*U*^11^	*U*^22^	*U*^33^	*U*^12^	*U*^13^	*U*^23^
O1	0.0280 (5)	0.0261 (4)	0.0677 (7)	0.0017 (3)	0.0007 (4)	−0.0079 (4)
O2	0.0264 (4)	0.0246 (4)	0.0426 (5)	0.0066 (3)	0.0043 (3)	−0.0004 (3)
N1	0.0270 (5)	0.0304 (5)	0.0283 (5)	0.0045 (4)	0.0007 (4)	0.0036 (4)
N2	0.0324 (5)	0.0247 (5)	0.0304 (5)	0.0045 (4)	0.0059 (4)	0.0012 (4)
N3	0.0324 (5)	0.0245 (5)	0.0303 (5)	0.0051 (4)	0.0064 (4)	0.0019 (4)
N4	0.0401 (6)	0.0282 (5)	0.0282 (5)	0.0031 (4)	0.0064 (4)	0.0018 (4)
C1	0.0281 (6)	0.0389 (6)	0.0290 (6)	0.0067 (5)	0.0029 (4)	0.0080 (5)
C2	0.0381 (7)	0.0427 (7)	0.0303 (6)	0.0165 (5)	0.0098 (5)	0.0089 (5)
C3	0.0479 (8)	0.0306 (6)	0.0320 (6)	0.0125 (5)	0.0098 (5)	0.0021 (5)
C4	0.0373 (7)	0.0275 (6)	0.0319 (6)	0.0046 (5)	0.0072 (5)	0.0017 (5)
C5	0.0271 (6)	0.0260 (5)	0.0256 (6)	0.0060 (4)	0.0008 (4)	0.0033 (4)
C6	0.0254 (6)	0.0254 (5)	0.0252 (5)	0.0047 (4)	−0.0007 (4)	0.0016 (4)
C7	0.0249 (6)	0.0237 (5)	0.0287 (6)	0.0026 (4)	0.0005 (4)	0.0028 (4)
C8	0.0245 (5)	0.0238 (5)	0.0260 (5)	0.0048 (4)	−0.0018 (4)	0.0017 (4)
C9	0.0261 (6)	0.0241 (5)	0.0258 (5)	0.0046 (4)	0.0000 (4)	0.0023 (4)
C10	0.0305 (6)	0.0232 (5)	0.0273 (6)	0.0074 (4)	0.0031 (4)	0.0049 (4)
C11	0.0294 (6)	0.0261 (5)	0.0339 (6)	0.0076 (4)	0.0024 (5)	0.0040 (5)
C12	0.0304 (7)	0.0352 (6)	0.0436 (7)	0.0084 (5)	0.0089 (5)	0.0090 (5)
C13	0.0460 (8)	0.0393 (7)	0.0382 (7)	0.0124 (6)	0.0165 (6)	0.0066 (5)
C14	0.0496 (8)	0.0340 (6)	0.0312 (6)	0.0060 (6)	0.0102 (5)	0.0009 (5)
C15	0.0256 (6)	0.0221 (5)	0.0253 (5)	0.0032 (4)	0.0042 (4)	0.0035 (4)
C16	0.0222 (6)	0.0258 (5)	0.0307 (6)	0.0050 (4)	0.0019 (4)	0.0038 (4)
C17	0.0232 (6)	0.0245 (5)	0.0313 (6)	0.0014 (4)	0.0001 (4)	0.0014 (4)
C18	0.0249 (6)	0.0218 (5)	0.0268 (5)	0.0033 (4)	0.0039 (4)	0.0031 (4)
C19	0.0226 (6)	0.0263 (5)	0.0333 (6)	0.0051 (4)	0.0007 (4)	0.0029 (4)
C20	0.0248 (6)	0.0251 (5)	0.0336 (6)	0.0021 (4)	−0.0021 (4)	−0.0004 (4)
C21	0.0252 (6)	0.0253 (5)	0.0299 (6)	0.0045 (4)	0.0015 (4)	0.0026 (4)
C22	0.0339 (7)	0.0291 (6)	0.0484 (8)	0.0116 (5)	0.0040 (6)	−0.0034 (6)

### Geometric parameters (Å, º)

**Table:**

O1—C21	1.2049 (14)	C9—C10	1.4876 (16)
O2—C21	1.3342 (15)	C10—C11	1.3914 (17)
O2—C22	1.4501 (14)	C11—C12	1.3877 (18)
N1—C1	1.3411 (16)	C11—H11	0.994 (15)
N1—C5	1.3461 (15)	C12—C13	1.3833 (19)
N2—C6	1.3366 (15)	C12—H12	0.996 (16)
N2—N3	1.3407 (14)	C13—C14	1.389 (2)
N3—C9	1.3375 (14)	C13—H13	0.989 (18)
N4—C14	1.3388 (17)	C14—H14	0.968 (16)
N4—C10	1.3429 (15)	C15—C16	1.3909 (15)
C1—C2	1.3854 (18)	C15—C20	1.3926 (16)
C1—H1	0.998 (16)	C16—C17	1.3861 (15)
C2—C3	1.3804 (19)	C16—H16	0.967 (15)
C2—H2	0.979 (18)	C17—C18	1.3899 (16)
C3—C4	1.3837 (18)	C17—H17	0.954 (15)
C3—H3	0.990 (16)	C18—C19	1.3946 (15)
C4—C5	1.3909 (16)	C18—C21	1.4911 (15)
C4—H4	0.962 (17)	C19—C20	1.3851 (16)
C5—C6	1.4869 (16)	C19—H19	0.967 (15)
C6—C7	1.3997 (15)	C20—H20	0.987 (16)
C7—C8	1.3759 (16)	C22—H22A	1.000 (18)
C7—H7	0.975 (15)	C22—H22B	0.964 (19)
C8—C9	1.4151 (16)	C22—H22C	0.959 (19)
C8—C15	1.4864 (14)		
			
O2···C3^i^	3.4089 (16)	N4···H14^viii^	2.931 (18)
O1···H12^ii^	2.765 (16)	C1···C6^iii^	3.4447 (17)
O1···H22A	2.573 (18)	C1···C7^iii^	3.5272 (17)
O1···H22B	2.660 (18)	C2···C17^ix^	3.4292 (18)
O1···H22C^iii^	2.537 (19)	C3···C3^x^	3.5516 (18)
O1···H22B^iv^	2.598 (18)	C6···C11^iii^	3.3751 (16)
O1···H17	2.611 (14)	C10···C20	3.3022 (16)
O2···H17^v^	2.773 (13)	C11···C22^vii^	3.4630 (18)
O2···H19	2.462 (14)	C14···C17^viii^	3.4960 (18)
N1···C9^iii^	3.4168 (15)	C14···C16^viii^	3.4987 (18)
N2···C1^v^	3.4404 (16)	C1···H19^i^	2.926 (16)
N4···C20	3.2459 (16)	C6···H11^iii^	2.924 (15)
N4···C15	2.8825 (15)	C7···H1^v^	2.988 (17)
N4···C16	3.4362 (16)	C11···H22A^vii^	2.964 (18)
N1···H20^iii^	2.750 (15)	C16···H14^viii^	2.891 (17)
N1···H7	2.553 (14)	C17···H2^ix^	2.898 (18)
N2···H4^vi^	2.710 (17)	C18···H2^ix^	2.822 (18)
N2···H4	2.511 (17)	H3···H11^vi^	2.53 (2)
N3···H11	2.644 (13)	H4···H4^vi^	2.43 (3)
N3···H22A^vii^	2.713 (17)	H7···H20^iii^	2.43 (2)
N3···H3^vi^	2.644 (18)	H16···H19^iii^	2.57 (2)
			
C21—O2—C22	115.72 (9)	C10—C11—H11	120.0 (9)
C1—N1—C5	116.73 (10)	C13—C12—C11	118.62 (12)
C6—N2—N3	119.41 (9)	C13—C12—H12	121.9 (9)
C9—N3—N2	120.51 (9)	C11—C12—H12	119.5 (9)
C14—N4—C10	117.22 (11)	C12—C13—C14	118.80 (12)
N1—C1—C2	123.85 (11)	C12—C13—H13	121.2 (10)
N1—C1—H1	114.9 (9)	C14—C13—H13	120.0 (10)
C2—C1—H1	121.3 (9)	N4—C14—C13	123.44 (12)
C3—C2—C1	118.61 (11)	N4—C14—H14	115.5 (10)
C3—C2—H2	123.1 (10)	C13—C14—H14	121.0 (9)
C1—C2—H2	118.3 (10)	C16—C15—C20	119.63 (10)
C2—C3—C4	118.80 (12)	C16—C15—C8	120.52 (10)
C2—C3—H3	121.6 (10)	C20—C15—C8	119.84 (10)
C4—C3—H3	119.6 (10)	C17—C16—C15	120.28 (10)
C3—C4—C5	118.79 (12)	C17—C16—H16	120.5 (8)
C3—C4—H4	121.6 (10)	C15—C16—H16	119.1 (8)
C5—C4—H4	119.6 (10)	C16—C17—C18	119.99 (10)
N1—C5—C4	123.17 (11)	C16—C17—H17	120.6 (9)
N1—C5—C6	115.74 (10)	C18—C17—H17	119.4 (9)
C4—C5—C6	121.07 (10)	C17—C18—C19	119.84 (10)
N2—C6—C7	122.43 (10)	C17—C18—C21	118.84 (10)
N2—C6—C5	115.74 (10)	C19—C18—C21	121.31 (10)
C7—C6—C5	121.82 (10)	C20—C19—C18	120.00 (10)
C8—C7—C6	118.68 (10)	C20—C19—H19	121.2 (8)
C8—C7—H7	121.2 (8)	C18—C19—H19	118.8 (8)
C6—C7—H7	120.1 (8)	C19—C20—C15	120.17 (10)
C7—C8—C9	116.35 (10)	C19—C20—H20	121.7 (9)
C7—C8—C15	121.43 (10)	C15—C20—H20	118.2 (9)
C9—C8—C15	122.17 (10)	O1—C21—O2	123.69 (10)
N3—C9—C8	122.45 (10)	O1—C21—C18	124.18 (10)
N3—C9—C10	114.54 (10)	O2—C21—C18	112.13 (9)
C8—C9—C10	122.98 (10)	O2—C22—H22A	108.3 (9)
N4—C10—C11	123.19 (11)	O2—C22—H22B	109.6 (10)
N4—C10—C9	115.56 (10)	H22A—C22—H22B	111.1 (14)
C11—C10—C9	121.21 (10)	O2—C22—H22C	104.1 (10)
C12—C11—C10	118.71 (11)	H22A—C22—H22C	111.1 (14)
C12—C11—H11	121.3 (9)	H22B—C22—H22C	112.3 (15)
			
C6—N2—N3—C9	1.13 (16)	N3—C9—C10—C11	−37.56 (15)
C5—N1—C1—C2	−0.15 (17)	C8—C9—C10—C11	144.40 (12)
N1—C1—C2—C3	1.69 (19)	N4—C10—C11—C12	0.83 (18)
C1—C2—C3—C4	−1.34 (19)	C9—C10—C11—C12	178.28 (10)
C2—C3—C4—C5	−0.40 (19)	C10—C11—C12—C13	0.11 (18)
C1—N1—C5—C4	−1.75 (17)	C11—C12—C13—C14	−1.0 (2)
C1—N1—C5—C6	176.55 (10)	C10—N4—C14—C13	−0.2 (2)
C3—C4—C5—N1	2.05 (19)	C12—C13—C14—N4	1.1 (2)
C3—C4—C5—C6	−176.17 (11)	C7—C8—C15—C16	−64.14 (15)
N3—N2—C6—C7	−3.53 (17)	C9—C8—C15—C16	118.48 (12)
N3—N2—C6—C5	176.55 (9)	C7—C8—C15—C20	114.96 (12)
N1—C5—C6—N2	−162.08 (10)	C9—C8—C15—C20	−62.42 (15)
C4—C5—C6—N2	16.27 (16)	C20—C15—C16—C17	2.49 (17)
N1—C5—C6—C7	18.00 (16)	C8—C15—C16—C17	−178.41 (10)
C4—C5—C6—C7	−163.65 (11)	C15—C16—C17—C18	0.21 (17)
N2—C6—C7—C8	1.90 (17)	C16—C17—C18—C19	−2.31 (17)
C5—C6—C7—C8	−178.18 (10)	C16—C17—C18—C21	179.05 (10)
C6—C7—C8—C9	1.90 (16)	C17—C18—C19—C20	1.71 (17)
C6—C7—C8—C15	−175.63 (10)	C21—C18—C19—C20	−179.68 (11)
N2—N3—C9—C8	2.85 (17)	C18—C19—C20—C15	0.99 (18)
N2—N3—C9—C10	−175.20 (10)	C16—C15—C20—C19	−3.08 (17)
C7—C8—C9—N3	−4.30 (16)	C8—C15—C20—C19	177.81 (10)
C15—C8—C9—N3	173.21 (10)	C22—O2—C21—O1	2.69 (18)
C7—C8—C9—C10	173.58 (10)	C22—O2—C21—C18	−177.94 (10)
C15—C8—C9—C10	−8.91 (17)	C17—C18—C21—O1	21.65 (18)
C14—N4—C10—C11	−0.80 (18)	C19—C18—C21—O1	−156.97 (13)
C14—N4—C10—C9	−178.38 (11)	C17—C18—C21—O2	−157.72 (10)
N3—C9—C10—N4	140.07 (11)	C19—C18—C21—O2	23.66 (15)
C8—C9—C10—N4	−37.96 (15)		

Symmetry codes: (i) −*x*+1, −*y*+1, −*z*+1; (ii) *x*+1, *y*+1, *z*; (iii) *x*+1, *y*, *z*; (iv) −*x*+1, −*y*+2, −*z*+2; (v) *x*−1, *y*, *z*; (vi) −*x*+1, −*y*, −*z*+1; (vii) *x*, *y*−1, *z*; (viii) −*x*+1, −*y*+1, −*z*+2; (ix) −*x*+2, −*y*+1, −*z*+1; (x) −*x*+2, −*y*, −*z*+1.

### Hydrogen-bond geometry (Å, º)

*Cg*1 is the centroid of the pyridyl ring, *A* (N1/C1–C5).

**Table:**

*D*—H···*A*	*D*—H	H···*A*	*D*···*A*	*D*—H···*A*
C19—H19···*Cg*1^i^	0.967 (15)	2.876 (15)	3.5715 (13)	129.8 (12)
C22—H22*B*···O1^iv^	0.964 (19)	2.598 (19)	3.5407 (19)	165.8 (14)
C22—H22*C*···O1^v^	0.959 (19)	2.536 (19)	3.4924 (16)	174.8 (15)

Symmetry codes: (i) −*x*+1, −*y*+1, −*z*+1; (iv) −*x*+1, −*y*+2, −*z*+2; (v) *x*−1, *y*, *z*.
